# Quantitative *In Situ* Measurement of Estrogen Receptor mRNA Predicts Response to Tamoxifen

**DOI:** 10.1371/journal.pone.0036559

**Published:** 2012-05-11

**Authors:** Jennifer M. Bordeaux, Huan Cheng, Allison W. Welsh, Bruce G. Haffty, Donald R. Lannin, Xingyong Wu, Nan Su, Xiao-Jun Ma, Yuling Luo, David L. Rimm

**Affiliations:** 1 Department of Pathology, Yale University School of Medicine, New Haven, Connecticut, United States of America; 2 Advanced Cell Diagnostics, Hayward, California, United States of America; Northwestern University Feinberg School of Medicine, United States of America

## Abstract

**Purpose:**

Quantification of mRNA has historically been done by reverse transcription polymerase chain reaction (RT-PCR). Recently, a robust method of detection of mRNA utilizing *in situ* hybridization has been described that is linear and shows high specificity with low background. Here we describe the use of the AQUA method of quantitative immunofluorescence (QIF) for measuring mRNA in situ using *ESR1* (the estrogen receptor alpha gene) in breast cancer to determine its predictive value compared to Estrogen Receptor α (ER) protein.

**Methods:**

Messenger RNA for ER (*ESR1*) and Ubiquitin C (*UbC*) were visualized using RNAscope probes and levels were quantified by quantitative *in situ* hybridization (qISH) on two Yale breast cancer cohorts on tissue microarrays. *ESR1* levels were compared to ER protein levels measured by QIF using the SP1 antibody.

**Results:**

*ESR1* mRNA is reproducibly and specifically measurable by qISH on tissue collected from 1993 or later. *ESR1* levels were correlated to ER protein levels in a non-linear manner on two Yale cohorts. High levels of *ESR1* were found to be predictive of response to tamoxifin.

**Conclusion:**

Quantification of mRNA using qISH may allow assessment of large cohorts with minimal formalin fixed, paraffin embedded tissue. Exploratory data using this method suggests that measurement of *ESR1* mRNA levels may be predictive of response to endocrine therapy in a manner that is different from the predictive value of ER.

## Introduction

Despite the usefulness of ER as a predictive marker for endocrine therapy 50% of ER positive patients still recur, indicating a need for additional predictive biomarkers for endocrine therapy [1]. Genomic technologies have allowed the search for new potential biomarkers beyond the traditional protein-based Immunohistochemistry (IHC) markers to gene expression signatures using messenger RNA (mRNA) to provide prognostic or predictive information [2]. One such example is the Onco*type* DX assay that uses 21 genes to determine a recurrence score to quantify the risk of distant recurrence in tamoxifen-treated, lymph node negative, ER positive breast cancer [3], [4], [5]. These results suggest that assessment of mRNA levels may carry information regarding response to therapy that could be complementary or unique from the information conveyed by the assessment of protein expression.

Assessment of mRNA expression signatures allows for the comparison of thousands of genes at a time. As a result, mRNA expression-based signatures, like the Agendia Mammaprint test and the PAM50 have shown that better patient stratification can be achieved by looking at many genes [6], [7]. However, recently, Paik and colleagues have suggested that even looking at the mRNA from a single gene could show predictive power [8]. This observation raises the concept of measurement of mRNA in the same way we measure protein, that is, using *in situ* methods. Recently a novel mRNA *in situ* hybridization (ISH) technique called RNAscope (Advanced Cell Diagnostics, Inc., Hayward, CA) has been developed that can be used to detect RNA transcripts on formalin-fixed paraffin embedded (FFPE) tissue [9], [10], [11], [12], [13]. This method provides the opportunity to measure mRNA in large collections of FFPE tissue where conventional methods of obtaining mRNA would be limiting. However, in order for *in situ* methods to have value similar to RT-PCR, the analysis must be combined with a quantitative tool. Here we modified the AQUA method for quantitative measurement of protein to combine it with the RNAscope method to quantify ER mRNA (*ESR1*) *in situ* and to compare to ER protein levels determined by quantitative immunofluorescence (QIF) on two breast cancer cohorts.

## Results

### RNAscope Assay Validation

**Figure 1:**
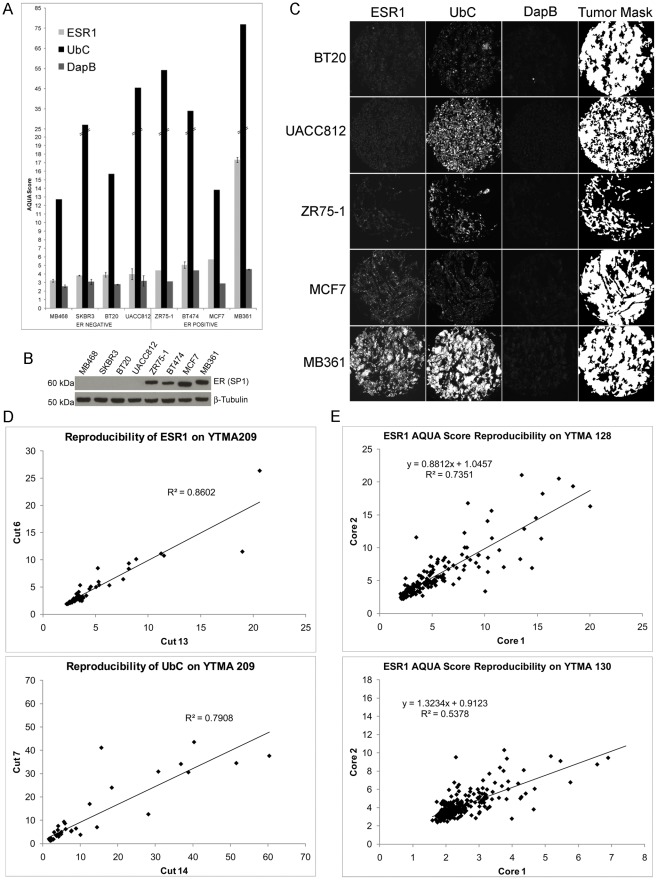
Validation of the RNAscope assay. (A) Average AQUA score distributions of the RNAscope assay for *ESR1*, *UbC*, and *DapB* performed on serial sections of the control array (YTMA 188) are shown in order of increasing *ESR1.* Error bars represent standard deviation for those cell lines where both cores were available for analysis. (B) 20 µg total cell lysate from MDA-MB-468, SKBR3, BT20, UACC812, ZR75-1, BT474, MCF7 and MDA-MB-231 were probed with ER SP1 antibody by Western blot. β-Tubulin served as a loading control. (C) Representative images are shown for *ESR1* negative cell lines BT20 and UACC812 and *ESR1* positive cell lines ZR75-1, MCF7 and MDA-MB-361 with the corresponding *UbC* positive control, *DapB* negative control, and tumor mask compartment generated using cytokeratin. (D) Reproducibility of the assay between serial sections of the same TMA core is shown on the breast index array (YTMA 209) for *ESR1* and *UbC.* (E) Reproducibility of *ESR1* between 2 patient cores on YTMA 128 (top) and YTMA 130 (bottom).

The RNAscope® assay for *ESR1*, *UbC*, and *DapB* was first performed on serial sections of a control TMA containing a panel of ER positive and ER negative breast cancer cell lines in 2-fold redundancy and quantified using AQUA (Fig. 1a). The method of quantitative in situ hybridization (qISH) gave AQUA scores for *ESR1* ranging from 3–17 with threshold for positivity by the assay corresponding to an AQUA score of 4. As expected, MDA-MB-468, SKBR3, BT20, and UACC812 lines were all negative and ZR75-1, BT474, MCF7, and MDA-MB-361 lines were positive for *ESR1*. The same cell lines were negative or positive respectively for ER protein expression as determined by Western blot with SP1 (Fig. 1b). Representative images for *ESR1*, *UbC*, and *DapB* as well as the tumor mask generated by cytokeratin staining are shown in Fig. 1c.

The qISH of *ESR1* and *UbC* by this assay was highly reproducible (R^2^ of 0.86 and 0.79 respectively) on serial sections of a control array with 71 breast cancer cases (YTMA 209) run in 2 independent experiments (Fig. 1d). Additionally, AQUA scores for *ESR1* were compared for 2 independent cores from the YTMA 128 cohort (R^2^ of 0.74) and from the YTMA 130, (R^2^ of 0.54, Fig. 1e) illustrating the level of heterogeneity of *ESR1* expression between different cores of the same tumor.

### mRNA Quality for RNAscope on FFPE Tissue is Dependent on Tissue Age

**Figure 2:**
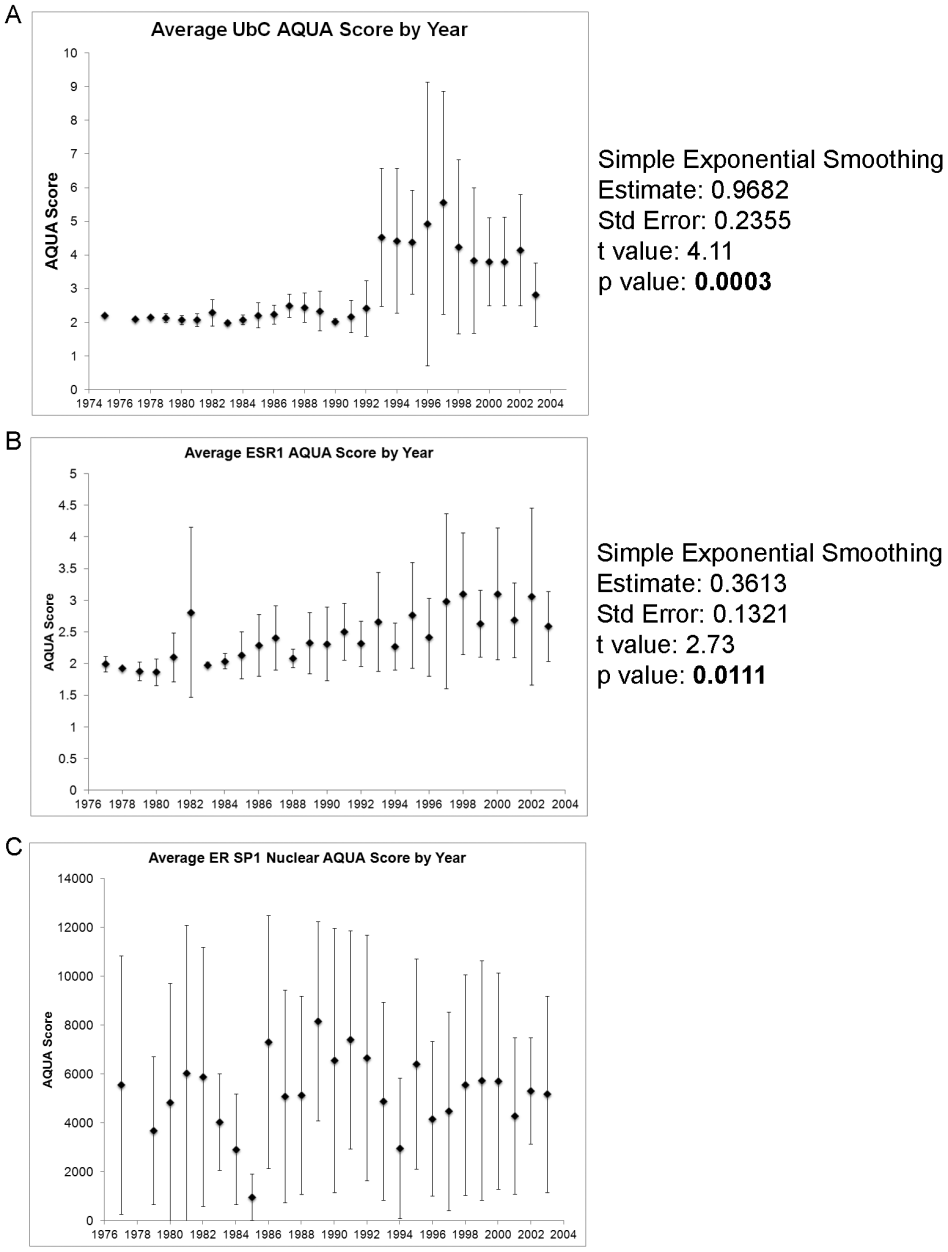
mRNA quality is dependent on tissue age. (A) Average AQUA scores for the *UbC* positive control by year. Time series testing after simple exponential smoothing demonstrates a statistically significant upward trend with an obvious split at 1993 (p = 0.0003). (B) Average AQUA scores by year for *ESR1* are shown only for patients with positive Path ER protein status. Time series testing after simple exponential smoothing demonstrates a statistically significant upward trend (p = 0.0111). (C) Average nuclear AQUA scores by year for are shown ER protein determined with SP1 only for patients with positive Path ER protein status and demonstrate no significant trend over time. Error bars represent standard deviation.

Previous reports have shown that RNA quality extracted from FFPE tissue is affected by the duration of FFPE tissue block storage [14]. To determine the patient tissue viable for analysis on YTMA 130, AQUA scores for *UbC* (positive control probe with expression expected in every tumor) were averaged by year. A time series test showed a significant increase in AQUA score (p = 0.0003) with an evident breakpoint at 1993 (Fig. 2a). A similar analysis for *ESR1* on ER positive cases from YTMA 130 identified also showed a significant increasing trend (p = 0.0111) with higher AQUA scores seen in the more recent the tissue samples (Fig. 2b). Therefore only cases from 1993 or later were included in subsequent analysis and are identified as the YTMA 130 Subset. There were no statistically significant differences in the clinicopathological characteristics between the complete cohort and YTMA 130 Subset (Table 1). Comparison of the average AQUA score of ER protein in ER positive cases demonstrated no trend from 1975–2003 (Fig. 2c).

**Table 1:** Cohorts characteristics and Post-1993 Subset from YTMA-130.

Characteristic	YTMA 128	YTMA 130	YTMA 130 Subset	
	No. (%)	No. (%)	No. (%)	p-value
All patients	238	524	226	
Age, years				
<50	75 (31.5)	152 (29.0)	73 (32.3)	
≥50	147 (61.8)	293 (55.9)	153 (67.7)	0.3151
Unknown	16 (6.7)	79 (15.1)	0 (0)	
Nodal status				
Positive	72 (30.3)	72 (13.7)	42 (18.6)	
Negative	151 (63.4)	253 (48.3)	146 (64.6)	0.4804
Unsampled/unknown	15 (6.3)	199 (38.0)	38 (16.8)	
Tumor size				
<2 cm	143 (60.1)	267 (51.0)	145 (64.2)	
2–5 cm	72 (30.3)	124 (23.7)	69 (30.5)	0.4469
>5 cm	10 (4.2)	2 (0.4)	0 (0)	
Unknown	13 (5.5)	131 (25.0)	12 (5.3)	
ER (IHC)				
Positive (1–3)	162 (68.1)	220 (42.0)	125 (53.3)	
Negative (0)	39 (16.4)	169 (32.3)	87 (38.5)	0.4823
Unknown	37 (15.5)	135 (25.8)	14 (6.2)	
PgR (IHC)				
Positive (1–3)	142 (59.7)	37 (7.1)	28 (12.4)	
Negative (0)	59 (24.8)	349 (66.6)	182 (80.5)	0.0804
Unknown	37 (15.5)	138 (23.3)	16 (7.1)	
Her2 (IHC)				
Positive (2–3)	56 (23.5)	39 (7.4)	26 (11.5)	
Negative (0–1)	140 (58.8)	344 (65.6)	186 (82.3)	0.2179
Unknown	42 (17.6)	141 (26.9)	14 (6.2)	
Follow-up, months				
Median	49	81	64.5	
Range	1–340	2–327	3–169	

Abbreviations: ER, estrogen receptor; IHC, immunohistochemistry; PgR, progesterone receptor; YTMA, Yale tissue microarray (cohort).

### Correlation Between ER mRNA and ER Protein

**Figure 3:**
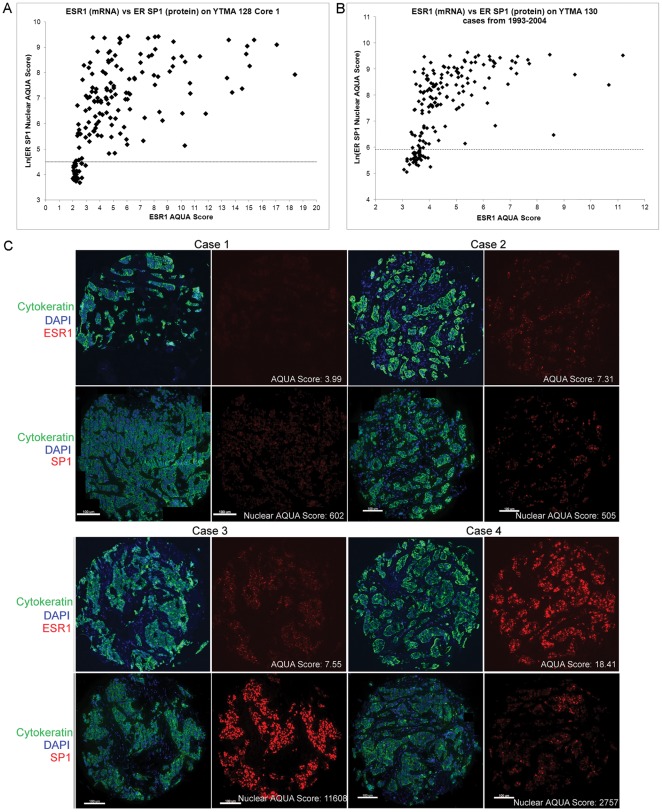
Comparison between *ESR1* and ER protein on Yale breast cancer cohorts. The natural log of the nuclear ER AQUA score determined by QIF using SP1 is shown on the y-axis and the AQUA score for *ESR1* determined by qISH is on x-axis for YTMA 128, n = 167 with scores for both SP1 and *ESR1* (A) and YTMA 130 1993–2005 Subset, n = 195 with scores for both SP1 and *ESR1* (B). The dotted line represents the threshold for ER protein positivity. (C) Representative images from 4 cases on YTMA 128 for cytokeratin (green), DAPI (blue), and *ESR1* or ER SP1 (red).

The qISH assay allows comparison of the level of ER mRNA to ER protein both quantified on a continuous scale using AQUA on serial sections of the breast cancer cohorts. The YTMA-128 cohort contained cases from 2002–2006 therefore all cases with scores for both ER SP1 (protein) and *ESR1* were used in the comparison, whereas only cases from the YTMA-130 Subset were used. The natural log of the nuclear SP1 protein AQUA scores was used to convert the scores to the same scale as the *ESR1* AQUA scores. Both cohorts demonstrate a positive, but non-linear correlation between ER mRNA and protein levels (Fig. 3a and b). Examples of the staining patterns observed from four cases on YTMA 128 are shown in Figure 3c. Case 1 demonstrates low levels of both ER mRNA and protein. Cases 2 and 3 have similar levels of ER mRNA, but Case 2 has low levels of protein whereas Case 3 has very high protein levels. Case 4 illustrates a less common example of high mRNA with moderate protein expression levels.

### ESR1 Predicts Response to Tamoxifen

**Figure 4:**
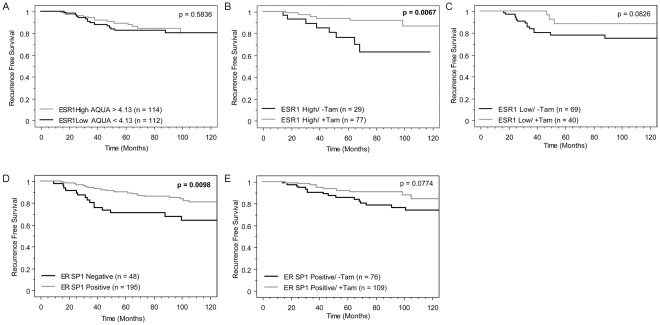
*ESR1* predicts response to tamoxifen on YTMA 130 Subset. Kaplan-Meier curves all show 10-year recurrence-free survival. (A) Cases from the YTMA 130 Subset were split by the median *ESR1* AQUA score and shows no prognostic value. (B) *ESR1* high (AQUA >4.13) cases from the YTMA 130 Subset were split by tamoxifen treatment status. *ESR1* high patients who received tamoxifen had a statistically significant reduced risk of recurrence. (C) *ESR1* low (AQUA <4.13) cases from the YTMA 130 Subset were split by tamoxifen treatment status and show no statistically significant trend. (D) ER positivity on YTMA 130 determined by QIF with SP1 is prognostic. (E) ER positive cases from YTMA 130 were split by tamoxifen treatment status. Patients who received tamoxifen had a reduced risk of recurrence trending towards significance. All p values were calculated using the log-rank test.

The *ESR1* AQUA scores from the 2 cores of YTMA 130 Subset (from 1993 and later) were averaged and the median AQUA score was used to define *ESR1* high and *ESR1* low patient populations. Kaplan-Meier analysis using recurrence free survival showed no prognostic value for *ESR1* (Fig. 4a). As previously described [15], ER positivity determined by QIF for ER protein expression is prognostic, where ER positive patients have a higher probability for recurrence free survival, p = 0.0098 (Fig. 4d). Interestingly, *ESR1* high patients treated with tamoxifen have significantly better (p = 0.0067) recurrence free survival compared to *ESR1* high patients who did not receive tamoxifen, indicating *ESR1* status is predictive for response to endocrine therapy (Fig. 4b). This was not seen in the *ESR1* low patient population (Fig. 4c). ER positivity determined by protein expression trended towards prediction of response to endocrine therapy, but did not reach statistical significance (Fig. 4e). When the variables were combined in a Cox proportional hazards multivariate analysis ER protein expression remained significant independent of age, tumor size, nodal status, PgR, HER2, and *ESR1* status (Table 2). A Cox model with just ER and *ESR1* was similar where ER was significant and independent of *ESR1* (p = 0.0028, Table 3).

**Table 2:** Multivariate Analysis for YTMA 130 Subset.

Variable (n = 126)	HR (95% CI)	p-value
Age		
<50	1.00	
>50	0.576 (0.20–1.63)	0.2983
Tumor Size		
<2 cm	1.00	
2–5 cm	1.338 (0.46–3.92)	0.2314
Nodal Status		
Negative	1.00	
Positive	3.629 (1.17–11.24)	**0.0255**
PgR		
Negative	1.00	
Positive	1.179 (0.25–5.67)	0.8375
Her2		
Negative	1.00	
Positive	1.437 (0.48–4.32)	0.3371
ER (SP1)		
Nuclear AQUA <361	1.00	
Nuclear AQUA >361	0.14 (0.04–0.539)	**0.0043**
ER (*ESR1*)	1.331 (0.83–2.13)	0.2314

Abbreviations: ER, estrogen receptor; IHC, immunohistochemistry; PgR, progesterone receptor.

**Table 3:** Multivariate Analysis of ER protein and ER mRNA for YTMA 130 Subset.

Variable (n = 126)	HR (95% CI)	p-value
ER (SP1)		
Nuclear AQUA <361	1.00	
Nuclear AQUA >361	0.169 (0.05–0.54)	**0.0028**
ER (*ESR1*)	1.360 (0.91–2.04)	0.1372

Abbreviation: ER, estrogen receptor.

## Discussion

In this work, we show that the RNAscope method of qISH is specific for *ESR1* and can be combined with the AQUA method of analysis to measure the levels of mRNA. The qISH approach also allows assessment of reproducibility between runs and between histospots on a TMA. Unlike more common RT-PCR based methods for measurement of mRNA, the qISH method allows assessment in epithelial tissue only and conserves potential spatial information while normalizing for the amount of epithelium present in the specimen. Since the method does not require extraction or microdissection, it allows large cohort analysis on minimal tissue specimens (a single histospot on a TMA). We believe these advantages could broadly increase the specimen range of tissues available for mRNA analysis.

Using this qISH assay, we observed a non-linear relationship between ER mRNA and protein (Fig. 3). While previous work has largely shown proportional relationships between ER protein and ESR1 mRNA [8], [16], [17], [18]; in both cohorts we found a proportional, but non-linear relationship. Specifically, cases with relatively low levels of *ESR1* (AQUA score <5) demonstrated a wide range of ER protein expression whereas the majority of cases with the highest *ESR1* levels were also among the highest expressers of ER protein. We did not observe a single case with high levels of *ESR1* that was negative for ER protein expression. However there were cases with high ER protein expression that were right around the threshold of detection for *ESR1* by this assay. We believe these observations reflect differences in stability of ER protein versus *ESR1* mRNA. Future studies will be required to test this hypothesis.

While ER protein levels have been shown many times to be both predictive and prognostic, less data was available on mRNA. The recent reporting of *ESR1* mRNA as part of the Onco*type* DX test has raised the issue of predictive value since the test is now in broad clinical use. Members of the Genomic Health group and others recently reanalyzed the B14 data to show that, in fact, mRNA for *ESR1* is predictive when measured by their RT-PCR assay [8]. This work confirms that observation, but extends it by illustrating the non-linear relationship between mRNA and protein. We also show that that prognostic value of ER is independent of the mRNA levels in multivariate models.

While these observations are provocative, the study has a series of limitations that must be considered. Perhaps the most significant is that the outcome analysis could only be performed in a single cohort that was further limited by the fact that only cases after 1993 could be used. This cohort was also limited by the fact that is a retrospective collection rather than a prospective trial, and as such, treatment was not controlled and homogeneous. Although a second cohort was assessed, which supported the conclusion of a non-linear relationship; no survival analysis could be performed on this cohort as the collection is too recent for meaningful follow-up. Another limitation of this study is the choice of cut-point that defined *ESR1* high versus *ESR1* low patient populations. We chose the median as an objective cut-point even though it may not be the most biologically relevant. Finally, the analyses were done on tissue from a single site (Yale New Haven Hospital). While we show that the data prior to 1993 shows degradation, we cannot conclude that is a function of tissue age since it is possible there was a change in some laboratory reagent that year that resulted in a change of stability of the mRNA is the FFPE material. Finally, this material, like all historical FFPE material is subject to pre-analytic variation. We are unaware of any systematic assessment of the effects of pre-analytic variation on mRNA measured *in situ*. We look forward to testing the assay on material from other sites and on material that has controlled pre-analytic variables (Neumeister et al, under review).

In summary, we show that qISH can be used to reproducibly and quantitatively measure mRNA in TMA sections. The analysis of *ESR1* and ER on the same TMA histospots suggests a non-linear relationship and that *ESR1* is predictive of response to endocrine therapy. However, given the retrospective cohorts used in these discovery-based studies, this work must be considered exploratory. We look forward to applying this technology to large multi-institutional cooperative group studies.

## Materials and Methods

### Patient Cohorts

Two tissue microarray (TMA) cohorts of archival breast cancer samples from Yale were used in this study. The Yale Sentinel Node Cohort, called YTMA 128 (patients diagnosed from 2002–2006, n = 238) was accrued by Dr. Donald Lannin. An independent and non-overlapping cohort, called YTMA 130, from patients diagnosed from 1976–2005, (n = 524) was accrued by Dr. Bruce Haffty. Clinicopathologic characteristics of both cohorts are found in Table 1.

### RNA in Situ Hybridization

ISH for ER mRNA was performed using the RNAscope FFPE assay kit according to the manufacturer’s instructions with modifications for fluorescence detection of transcripts using Cy5-tyramide. Briefly, 5 µm thick TMA sections were treated with heat and protease digestion followed by hybridization with a mixture containing target probes to *ESR1*, the housekeeping gene *Ubiquitin C* (*UbC*) as a positive control or the bacterial gene *DapB* as a negative control. *ESR1* or *UbC* specific hybridization signals were detected with Cy5-tyramide. Sections were the incubated with 0.3% bovine serum albumin (BSA) in 0.1 mol/L of Tris-buffered saline (triethanolamine-buffered saline, pH 8) for 30 minutes at room temperature followed by incubation with a wide-spectrum rabbit anti-cow cytokeratin antibody (Z0622 1∶100, DAKO Corp, Carpinteria, CA) in BSA/tris-buffered saline for 1 hour at room temperature. The cytokeratin signal was detected with Alexa 546 conjugated goat anti-rabbit (1∶100, Molecular Probes, Eugene, OR) incubated for 1 hour at room temperature. Slides were then mounted using ProlongGold plus 4,6-diamidino-2-phenylindole (DAPI).

### Western Blot Analysis

Cells were harvested in NP-40 lysis buffer (1% NP-40, 20 mM Tris-HCl, 137 mM NaCl, 10% glycerol, 2 mM EDTA, 1 mM DTT, 1 x Complete midi-EDTA protease inhibitor cocktail (Roche Diagnostics, Indianapolis, IN), 1 mM sodium orthovanadate). Lysate concentrations were measured using Bio-Rad (Hercules, CA) reagent, and lysate was loaded onto NuPAGE 4–12% Bis-tris gels and transferred to 0.2 µm nitrocellulose membrane (Bio-Rad). Membranes were blocked in 5% milk, TBS, and 1% Tween for 1 hour at room temperature. Primary antibody SP1 (1∶1000, Thermo Scientific, Rockford, IL) was added to TBS/Tween and incubated overnight at 4**°**C. Primary antibody β-Tubulin (1∶1000, Cell Signaling Technology, Beverly, MA) was added to TBS/Tween and incubated for 1 hr at room temperature. Membranes were washed with TBS/Tween and then incubated with horseradish peroxidase–conjugated goat anti-rabbit secondary antibody (Jackson ImmunoResearch Laboratories, Inc., West Grove, PA) at a dilution of 1∶5,000 for 1 hour at room temperature. Bands were detected using SuperSignal West Pico Substrate (Pierce, Rockford, IL) and exposed to film.

### Immunofluorescence Staining

TMAs were stained with cytokeratin, DAPI and ER (SP1 1∶1000) using a standard protocol developed in our laboratory and recently described [15], [19].

### Quantitative Analysis

The AQUA® method of quantitative immunofluorescence (QIF) is a method that allows exact and objective measurement of fluorescence intensity within a defined tumor area, as well as within subcellular compartments, as described elsewhere [20]. Briefly, a series of monochromatic high-resolution images were captured using an Olympus AX-51 epifluorescent microscope using a previously described algorithm for image collection [20]. For each histospot an in and out-of-focus image were obtained for each fluorescence channel, DAPI (nuclei), Alexa 546 (cytokeratin), or Cy5 (target probe). A tumor mask was created by binarizing the cytokeratin signal and target probe expression was quantified only in the tumor. AQUA scores were calculated for a given target within the tumor mask by dividing the signal intensity by the area of the tumor mask within the histospot. Patient sample histospots with less than 5% tumor, determined by the percentage area positive for cytokeratin were excluded from the analysis.

### Statistical Analysis

Time series testing followed by simple exponential smoothing was done using JMP 9 (SAS Institute). All other statistical testing was done using StatView 5.0 (SAS Institute) software. Kaplan-Meier survival analyses were performed for recurrence free survival and statistical significance was assessed by using the log-rank test. Multivariate analysis using the Cox proportional hazards model was performed to assess the independent prognostic significance of *ESR1* on recurrence free survival.
